# Development of Microfluidic Stretch System for Studying Recovery of Damaged Skeletal Muscle Cells

**DOI:** 10.3390/mi9120671

**Published:** 2018-12-18

**Authors:** Wanho Kim, Jaesang Kim, Hyung-Soon Park, Jessie S. Jeon

**Affiliations:** 1 Department of Mechanical Engineering, Korea Advanced Institute of Science and Technology, Daejeon 34141, Korea; do3ob@kaist.ac.kr (W.K.); oyoki@kaist.ac.kr (J.K.); 2 KI HST, Korea Advanced Institute of Science and Technology, Daejeon 34141, Korea

**Keywords:** skeletal muscle cells, muscle damage, recovery model, mechanical stretch, microfluidics

## Abstract

The skeletal muscle occupies about 40% mass of the human body and plays a significant role in the skeletal movement control. Skeletal muscle injury also occurs often and causes pain, discomfort, and functional impairment in daily living. Clinically, most studies observed the recovery phenomenon of muscle by massage or electrical stimulation, but there are limitations on quantitatively analyzing the effects on recovery. Although additional efforts have been made within in vitro biochemical research, some questions still remain for effects of the different cell microenvironment for recovery. To overcome these limitations, we have developed a microfluidic system to investigate appropriate conditions for repairing skeletal muscle injury. First, the muscle cells were cultured in the microfluidic chip and differentiated to muscle fibers. After differentiation, we treated hydrogen peroxide and 18% axial stretch to cause chemical and physical damage to the muscle fibers. Then the damaged muscle fibers were placed under the cyclic stretch condition to allow recovery. Finally, we analyzed the damage and recovery by quantifying morphological change as well as the intensity change of intracellular fluorescent signals and showed the skeletal muscle fibers recovered better in the cyclic stretched condition. In total, our in situ generation of muscle damage and induction recovery platform may be a key system for investigating muscle recovery and rehabilitation.

## 1. Introduction

Skeletal muscle occupies most of the human body and plays a significant role in movement and maintaining the shape of the body. From these characteristics, skeletal muscle is frequently exposed to injury during activity, and muscle injuries are one of the most common traumas in sports [1,2]. There are two types of muscle injury; one is the shearing type in which rupture occurs in the muscle fiber, mysial sheath, and basal lamina, and the other is myofiber necrosis, called rhabdomyolysis [3,4]. The shearing type is more common in daily life and people experience this as a contusion, muscle strain, and laceration [4,5,6].

Once muscle trauma occurs, the injury site will undergo three distinct healing processes [6,7]: (1) Inflammatory phase, (2) proliferative phase, and (3) remodeling/maturational phase. More specifically, for two days after injury, the inflammatory phase begins, with the removal of necrotic muscle tissue by macrophages. From the third day, the quiescent satellite cells in the injured area are activated and developed into proliferative myoblasts. These myoblasts are compacted in cellular space, fuse themselves and enter into the differentiation process. After enough differentiation, scars are created through remodeling [7]. The timescale of the muscle healing process varies with injury degree. The repair process usually takes three weeks but may require several months for severe functional loss and structural damage. Moreover, this injury has a high probability to leave aftereffects. In other words, the patient would need rehabilitation after healing [7,8,9,10,11]. Overall, muscle injury occurs frequently and causes discomfort from pain, healing time, and aftereffect in routine. Thus, the development of effective therapy for muscle recovery is consistently demanded.

There are numerous contributions in biochemical aspects that promote the healing process or initiate regeneration of muscle using nutrients [12,13], nonsteroidal anti-inflammatory drugs [14,15,16], steroids [17,18,19,20], and growth factor medication [21,22,23,24,25]. In addition, there have been attempts both in vivo and in vitro to investigate the effects of different experimental approaches such as therapeutic ultrasound [26,27,28,29,30] or hyperbaric oxygen therapy [31,32,33] on damaged muscle recovery and application of mechanical stretch [9,10,11,34,35,36,37,38,39] on myoblast proliferation and differentiation. While clinical research offers advantages in physiological relevance, limitations remain, in that the cellular level analysis is difficult. In addition, those complex physiological interactions such as inner/extra-cellular, neural, and hemodynamic interactions, may have combined effects, and lead to more challenges in elucidating the causal relationship. Conventional in vitro studies allow investigation on myoblast proliferation and differentiation, but in situ generation of muscle damage and stretch induction recovery model was difficult in the hard plastic experimental setting. Recent advances in the development of the microfluidic platform, which are applicable on various cell types as well as tissues, can be utilized to overcome these limitations, and enable better biomimicry and application of different stimuli with quantitative analysis to establish the optimal recovery conditions [40,41,42,43,44,45,46,47,48].

For this end, we have developed a stretchable microfluidic system that allows in situ generation of damage and recovery models of skeletal muscle cells. Our experiment focuses on revealing the recovery relationship between mechanical stretch and damaged muscle cells, and we also make an effort to establish the platform for analyzing the stretching treatment of muscle recovery quantitatively. With the microfluidic system, we instigated the strain injury as a damage model with excessive stretch and chemical damage. Subsequently, we observed the effects of cyclic stretch in the healing process by comparing the injury marker with the static condition. Taken together, we were able to investigate the constructive effects of cyclic stretch on the maturational recovery phase of the damaged muscle cells.

## 2. Materials and Methods

### 2.1. PDMS Device Design and Fabrication

A single channel microfluidic device was fabricated by photolithography (Figure S1a). A mixture of polydimethylsiloxane (PDMS, Dow Corning, Midland, MI, USA) base and curing agent at a weight ratio of 15:1, which is suitable for stretching with 18% strain, was poured into the master and degassed by a vacuum desiccator [49,50,51]. A PDMS film was prepared to have the same weight ratio used to make the PDMS mold. They were then fully cured for 120 min in 80 °C dry oven (Figure S1b,c). An inlet and outlet of the device were made by a biopsy punch of 2 mm diameter. PDMS film and device mold were sterilized using an autoclave. They were then bonded by plasma treatment (Figure S1d). Pipette tips were cut in order to form reservoirs for the single channel devices and inserted into the inlet and outlet (Figure S1e). Immediately after bonding, extracellular matrix coating was performed with the growth factor reduced Matrigel (BD Biosciences, Franklin Lakes, NJ, USA) containing 95% (v/v) Dulbecco’s modified Eagle medium (DMEM) to prevent cell detachment from PDMS film (Figure S1f).

### 2.2. Cell Culture

The C2C12 (ATCC, Manassas, VA, USA) myoblast cells were cultured in growth medium (GM) which is DMEM supplemented with 10% heat-inactivated fetal bovine serum (Gibco, Grand Island, NY, USA), and 1% antibiotic-antimycotic (Gibco) at 37 °C and 5% CO_2_ in an incubator. Cells were subcultured when they reached 80–90% confluence and they were seeded into the prepared devices before the passage of cells reached 8. The cells were detached with 0.25% trypsin-EDTA (Gibco) and seeded in the cell suspension state. The density and volume of the suspension were 2.0 × 10^6^ cells/mL and 100 µL, respectively. After seeding, the cells were allowed 2 h in the growth medium (GM) to adhere to the microfluidic channel. To flush and induce myogenic differentiation, the GM was aspirated and then switched to differentiation medium (DM) which is DMEM supplemented with 2% horse serum (Gibco), and 1% antibiotic-antimycotic (Figure S1g). DM was changed every 12 h for 4 days to induce differentiation.

### 2.3. Stretcher Design

**Figure 1:**
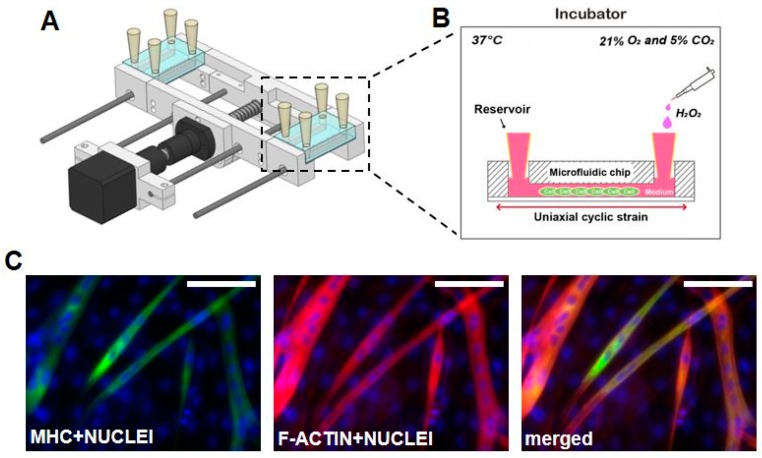
Stretchable microfluidic platform for inducing damage and recovery on skeletal muscle cells. (**a**) Schematic diagram of the lab-made stretcher with microfluidic chip and (**b**) detailed view of microfluidic chip is shown. The entire setup is placed in the incubator during experiments. The reservoir is filled with growth medium (GM) for the first 2 h after seeding and later supplemented with differentiation medium (DM). (**c**) Skeletal muscle differentiation is confirmed with immunofluorescence imaging of Myosin Heavy Chain (MHC) (green) and the presence of multi-nucleated C2C12 cells in microfluidic chip for 4 days after seeding. F-actin was stained with rhodamine-phalloidin (red), nuclei were stained with 4′,6-diamidino-2-phenylindole (DAPI) (blue), and merged shows the overlaid fluorescent image. (scale bar: 50 μm)

**Figure 2:**
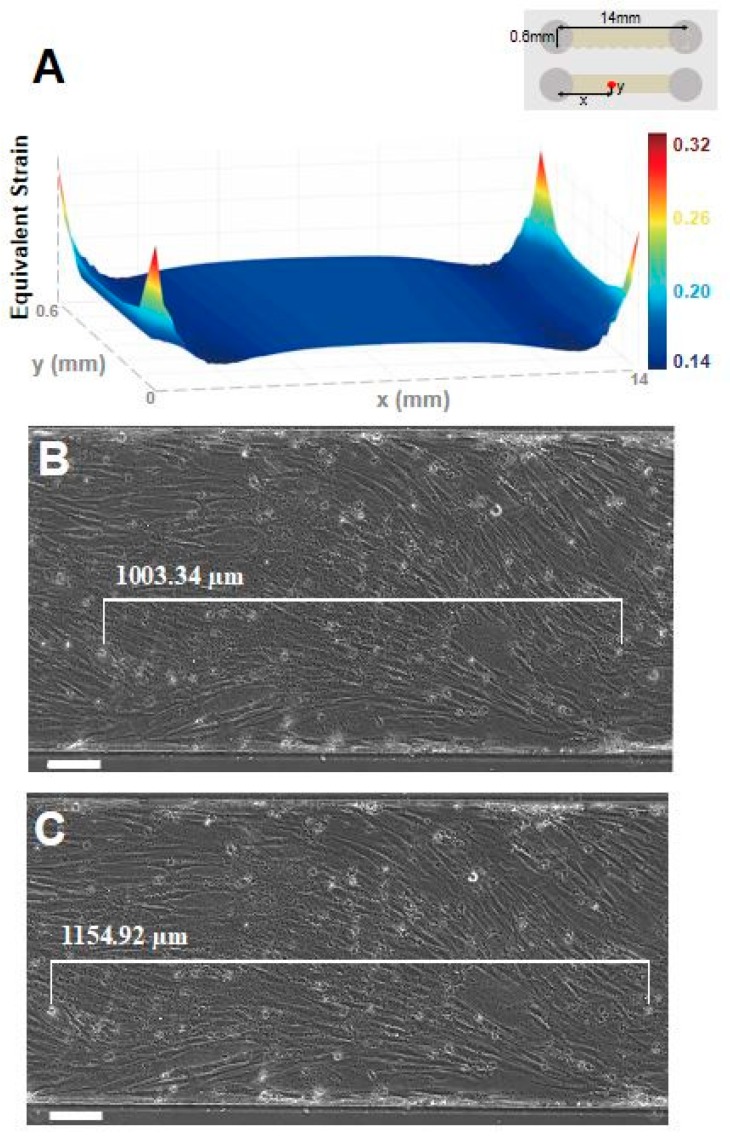
Stretcher simulation and validation on single channel device. (**a**) Simulation confirms about 15% equivalent elastic strain predominantly exists along the bottom surface of single channel device when 18% uniaxial stretch was given by the stretcher. The upper right inset shows the orientation of simulated microfluidic chip. Microscopic images of the device with cells (**b**) before and (**c**) after applying 18% elongation were used to measure the strain value applied to the cells. The distance between specific cells was measured before (1003.34 μm) and after (1154.92 μm) applying stretch, giving a total of 15.11% strain on the cell cultured device. (scale bar: 100 μm).

The in-house-made stretcher was fabricated with a 3D printer (Ultimaker, Geldermalsen, The Netherlands) using polylactic acid (PLA) material controlled by DRV8825 stepper motor driver (Pololu, Las Vegas, NV, USA) with design inspired from Wan et al. [52], and was used for applying cyclic stretch to the C2C12 cells (Figure 1). The positioning accuracy of the stretcher was validated, and the details are given in the supplemental material (Figure S2a). When elongating a single channel device using a stretcher, a strain simulation was performed to calculate the actual strain applied to the cells in the device by using a frontal solution program for finite element analysis in ANSYS R16.1 (ANSYS Inc., Canonsburg, PA, USA) (Figure 2). We simulated the stretching condition with 1.26 MPa elastic modulus of 15:1 (base:curing agent) PDMS, Poisson’s ratio of 0.5 in linearly elastic and isotropic deformation assumption (Table S1). Strain values obtained from simulation and experiment were compared in two different microfluidic chip designs to verify the validity of the simulation model (Figure 2, Figure S2b–d).

### 2.4. Creating Damage Model of Muscles Cells

After inducing myoblast differentiation with DM for four days, myotube damage was induced by two methods: (1) Chemical method using hydrogen peroxide (H_2_O_2_) to damage mitochondria and (2) mechanical method using the excessive stretch.

To induce chemical damage to the cells, hydrogen peroxide is commonly used as it damages mitochondria by allowing free electrons to leak during the respiration process, which increases the production of reactive oxygen species (ROS). ROS is known to act as an oxidative stress to cells, and therefore, its increased production induces damage to the cells [53]. In this experiment, H_2_O_2_ was diluted with DM to 1 mM and treated for 12 h to damage cells. Because H_2_O_2_ decomposes rapidly in the medium [32], it was treated once more 6 h after treatment.

To induce mechanical damage, the excessive stretch was applied. For normal muscle cells, less than 10% of stretch can be applied without serious injury [9]. However, 17% or greater stretch is considered to induce injury model [36,37,38]. In addition, in previous studies, cell viability was decreased, and ROS was increased and even apoptosis was observed when a stretch of more than 15% was applied to C2C12 cells [9]. Therefore, we used the stretcher to induce damage to cells by applying 18% stretch under the speed of 10 cycles/min for 12 h.

### 2.5. Recovery of Damaged Muscle Cells

To investigate the effect of cyclic stretch on cell recovery, we set the damage induced cells exposed to the cyclic stretch and compared them with the damage induced cells under static condition. The stretch condition is established using a stretcher in the incubator (BB15, Thermo Scientific, Waltham, MA, USA) with 95% air and 5% CO_2_ at 37 °C. Stretch parameters are given as follows: One cycle/30 min is given where one cycle consists of three sets [36]. One set consists of five repetitions of 10% stretch at 0.5 Hz, and the interval between sets is 30 s.

### 2.6. Staining and Image Processing

After inducing damage and establishing a recovery condition, intracellular ROS, Myosin Heavy Chain (MHC) (a biomarker for a maturity of skeletal muscle cell [54]), and 8-hydroxydeoxyguanosine (8-OHdG, a biomarker for oxidized DNA damage) staining were performed to quantify the damage and recovery of myotube. Intracellular ROS was imaged by introducing 10 μM CM-H_2_DCFDA (Invitrogen, Carlsbad, CA, USA) solution into the microchannel with cells after washing with PDMS. The devices were placed in a CO_2_ incubator (37 °C) for 30 min and washed with phosphate-buffered saline (PBS) twice before imaging with a fluorescent microscope.

For immunofluorescence staining, all medium in the devices was removed, and cells were fixed with 4% paraformaldehyde (PFA) for 15 min and later permeabilized with 0.1% Triton-X 100 solution for 5 min at room temperature. Afterward, the cells were treated with 3% Bovine Serum Albumin (BSA) solution for two hours for blocking. Mouse monoclonal myosin skeletal heavy chain antibody (Abcam, Cambridge, MA, USA) and mouse monoclonal DNA/RNA damage antibody (Abcam) were used for MHC and 8-OHdG staining at 1:100 and 1:500 dilution, respectively. Alexa Fluor 488 and 594 conjugated goat polyclonal secondary antibody (Abcam) was used at dilution 1:200. 4′,6-diamidino-2-phenylindole (DAPI) was diluted to 1:1000 with the secondary antibody for cell nuclei staining. Rhodamine phalloidin was also diluted to 1:40 to probe F-actin. All fluorescent images with MHC and 8-OHdG were obtained at a constant exposure time.

All fluorescent images were obtained using Axio Z1 (Carl Zeiss, Tokyo, Japan), and the images were analyzed using Image J (NIH) software. The fluorescent intensity of ROS and 8-OHdG in myotube was obtained by drawing linear segments in myotubes. In addition, since ROS signal can be found outside of cells, we merged the ROS image and phase contrast image to obtain the signal only in the cell. Mean intensity was obtained from each linear segment. Myotube diameter and MHC intensity data were measured from MHC fluorescent images. Parameter *n* indicates a number of cells counted for the analysis. All results were statistically analyzed with one-way analysis of variance (ANOVA) to compare the mean value of each experimental group and control group in damage confirmation and recovery comparison part.

## 3. Results

### 3.1. Confirmation of Myotube Formation in a Microfluidic Channel

C2C12 myoblasts were seeded in the microfluidic device and myogenic differentiation was induced by culturing in DM for 4 days. The differentiation of myoblasts into myotubes in the microfluidic channel was confirmed with MHC positive signal and the presence of multinucleated myotube formation as seen with the immunofluorescence staining of MHC and nuclei (Figure 1c).

### 3.2. Stretcher Simulation and Validation

In order to verify the performance of the stretcher, we compared the calculated strain from the simulation and the experimental strain values. The experimental strain values are measured as the microfluidic devices are elongated using a stretcher with attached cells. When the elongation is increased to 18% stretching, the simulation showed 15% strain throughout the bottom of the single channel device (Figure 2a). Furthermore, the experimental result also showed 15.1% strain on the bottom of the channel with the cells attached, which is comparable to the simulation results (Figure 2b).

### 3.3. Damage Confirmation

Damage on muscle cells in the microfluidic device was induced with two methods: Chemical and mechanical treatments. Muscle cells were treated chemically with hydrogen peroxide (H_2_O_2_, 1 mM) and mechanically by applying excessive stretch for 12 h. The induced damage was analyzed with four parameters, which are intracellular ROS intensity, myotube diameter, MHC intensity, and 8-OHdG (oxidized DNA damage) intensity.

**Figure 3:**
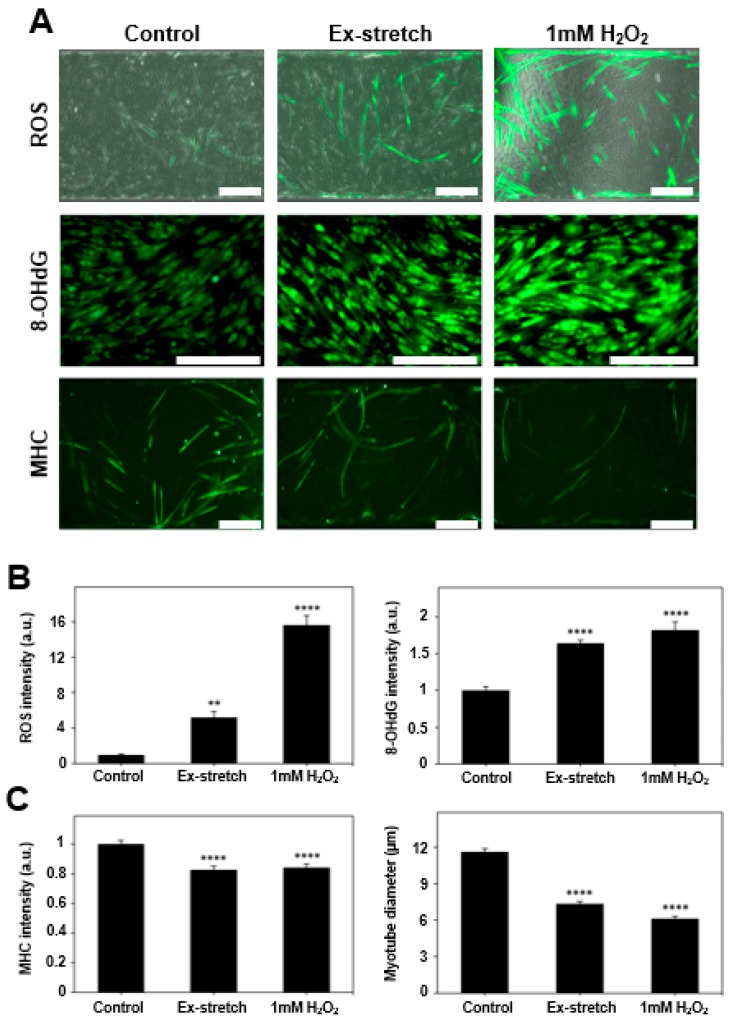
Confirmation of muscle cell damage. (**a**) Muscle cells are damaged with excessive stretch or H_2_O_2_ as shown with merged images of phase contrast and reactive oxygen species (ROS) fluorescent image, immunofluorescence images of 8-OHdG, and MHC. Control indicates the condition before damage (scale bar: 200 μm). (**b**) Increased fluorescent intensity of ROS (*n* > 54), 8-OHdG (*n* > 485), and (**c**) decrease in MHC (*n* > 173) fluorescence signals and myotube diameter confirm the muscle cell damage (all bars represent standard error mean. No star for *p* ≥ 0.05; * for *p* < 0.05; ** for *p* < 0.01; *** for *p* < 0.001; and **** for *p* < 0.0001 by one-way ANOVA for each group).

Contracting skeletal muscle produces free radicals composing ROS. When these free radicals maintain physiological levels, they play an important role in the regulation of gene expression, cell signaling pathways, and force production. However, prolonged and intense exercise can promote the generation of excessive free radicals, which leads to accumulation of ROS [34,35]. The ROS acts as oxidative stress to the cells, which can lead to cellular structural changes including DNA or RNA damage. Elevated ROS levels were also observed in a disease characterized by degeneration of muscle tissue [35]. Thus, ROS was used as a factor for confirming cellular damage. As a result of measuring ROS intensity in myotube, the intensity was higher in the excessive stretch (5.21 ± 0.67) (standard error of the mean) arbitrary unit, *p* < 0.01) and 1 mM H_2_O_2_ (15.6 ± 1.09, *p* < 0.0001) than in the control (1 ± 0.10) group before damage (Figure 3).

After damage induction, myotube diameter and MHC intensity, which are related to the functionality of the skeletal muscle [9,36], were measured to investigate the change of morphological characteristics and MHC amount of myotube. It was confirmed that the diameter was significantly decreased both (*p* < 0.0001) in the excessive stretch (7.34 ± 0.21 µm) and 1 mM H_2_O_2_ (6.13 ± 0.18 µm) compared to the undamaged control group (11.63 ± 0.26 µm). The intensity of MHC was also relatively reduced both (*p* < 0.0001) in the excessive stretch (0.827 ± 0.025) and 1mM H_2_O_2_ (0.843 ± 0.024) compared to the (1 ± 0.026) control group (Figure 3).

The intensity of 8-OHdG, an indicator of oxidative damage of DNA, was measured to investigate the DNA damage caused by elevated ROS in the two groups in which the damage was induced. As a result, the higher 8-OHdG intensity was observed both (*p* < 0.0001) in the excessive stretch (1.640 ± 0.046) and 1mM H_2_O_2_ (1.819 ± 0.113) compared to the undamaged control (1 ± 0.051) group (Figure 3).

### 3.4. Recovery Confirmation

**Figure 4:**
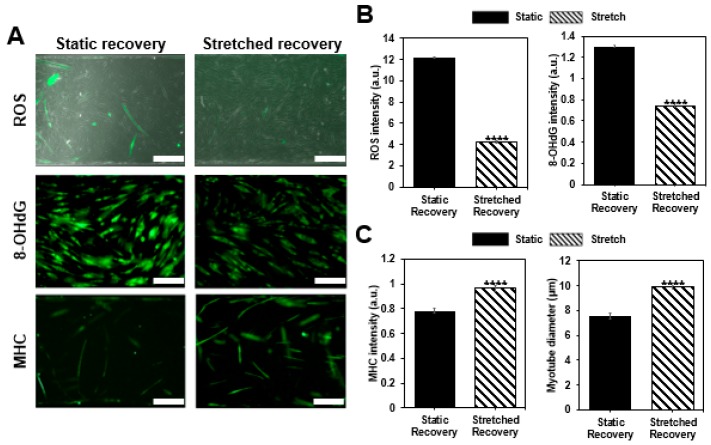
Confirmation of muscle recovery. (**a**) Once muscle cells are damaged by H_2_O_2_, the cells are allowed to recover in the static condition or with the cyclic stretch condition as shown with the merged images of phase contrast and ROS fluorescent image, immunofluorescence image of 8-OHdG, and MHC (scale bar: 200 μm). (**b**) Analysis of ROS (*n* > 54), 8-OHdG (*n* > 387), and (**c**) MHC (*n* > 172) intensity, as well as a morphological change of myotube by measuring diameter for each group, show the effect of stretch on recovery. Intensity values are normalized to the condition before the damage (all bars represent standard error mean. No star for *p* ≥ 0.05; * for *p* < 0.05; ** for *p* < 0.01; *** for *p* < 0.001; and **** for *p* < 0.0001 when tested with Student’s t-test for each group).

After inducing cellular damage with hydrogen peroxide (H_2_O_2_), static and stretch recovery conditions were maintained for 12 h. After 12 h, we compared two groups by confirming ROS fluorescence intensity, myotube diameter, MHC intensity, and DNA damage, as well as assessing the damage confirmation. All the intensity values are quantified using the arbitrary unit (a.u.) and normalized with the control group before damage. As a result, while both of the recovery conditions mostly showed improvement compared to the damaged condition, the given stretch condition showed enhanced recovery of damaged muscle cells (Figure 4). Both of the damage indicators, ROS level, and 8-OHdG, which have excessively increased after damage inducing (ROS: 15.6 ± 1.09, 8-OHdG: 1.819 ± 0.113), decreased with static recovery case (ROS: 12.1844 ± 1.06391, 8-OHdG: 1.30341 ± 0.01486), and further decreased with stretch recovery (ROS: 4.267 ± 0.52058, 8-OHdG: 0.74379 ± 0.0105), indicating that muscle cell conditions are improved compared to the damaged condition. Moreover, the two parameters for muscle cells, MHC intensity and myotube diameter, increased more in the stretch case (0.96399 ± 0.02237, 9.87048 ± 0.23759 μm) compared to the static case (0.78307 ± 0.02015, 7.54844 ± 0.19993 μm), and mostly show recovery from the damaged condition (0.843 ± 0.024, 6.13 ± 0.18 µm). Stretch seems to reduce the harmful factors, ROS, and 8-OHdG after damage. On the contrary, MHC expression and myotube formation were promoted by the stretch, i.e., stretch enhanced the muscle cell condition. Thus, we confirmed that the cyclic stretch has a positive effect on recovery.

## 4. Discussions

Chemical damage and mechanical damage were induced to skeletal muscle cells by using hydrogen peroxide and a lab-made stretcher platform. As a result, myoblasts produced abundant ROS, leading to DNA damage and apoptosis [39,55,56,57]. Therefore, ROS is not only an important parameter involved in the inflammatory reaction after injury but also an important indicator of damages to the DNA and membrane of the cell [37]. The 8-OHdG intensity, which is the predominant forms of free radical-induced oxidative lesions, was selected to confirm the DNA damage due to the elevated ROS level. Additionally, myotube diameter and MHC intensity were closely related to skeletal muscle function. Thus, changes observed on ROS, 8-OHdG, MHC intensity, and myotube diameter after damage could indicate the changes to their muscle functions.

Assuming the ROS level of the undamaged control group as the physiological level, the ROS intensity increased 5.2 times and 15.6 times, respectively, in the groups treated with mechanical and chemical damage inducing methods, and 8-OHdG intensity was also increased by 64.0% and 81.9% compared to the control group, respectively (Figure 3). Thus, it was confirmed that DNA damage is induced by the elevated ROS and that these two parameters indeed can establish the desired damage model. In previous studies, decreased cell viability and apoptosis were observed when ROS was increased by 5 times, and cell viability was reduced with the increase of mitochondrial fragmentation when hydrogen peroxide was treated [31,32]. In addition, the damage model could be further established by the measurements showing the decreased myotube diameter and lower MHC intensity in the damaged groups (Figure 3).

After confirming the induced damage, we gave the static and cyclic stretch condition for 12 h to compare the effect on recovery. It was reported that a similar stretch pattern improved the structural properties and functionality of human skeletal muscle cells [38]. As a result, the ROS intensity was 12 times higher in the static recovery condition (12.1844 ± 1.06391, *p* < 0.0001) compared to the non-damage condition (1 ± 0.10169), whereas only four times higher in the stretch condition (4.267 ± 0.52058, *p* < 0.0001). Likewise, 8-OHdG fluorescence intensity was compared to assess DNA damage, and stretch condition (0.74379 ± 0.0105, *p* < 0.0001) even showed 25.6% decrease compared to the non-damaged condition (1.000 ± 0.0101), while still high in the static condition (1.30341 ± 0.01486, *p* < 0.0001) (Figure 4a,b). In addition, the difference in MHC intensity between stretched case (0.96399 ± 0.02237, *p* < 0.0001) and non-damaged case (1 ± 0.02592) was less than 5%, while static case was lower than 20% (0.78307 ± 0.02015, *p* > 0.05) (Figure 4a,c). Furthermore, myotube diameter was measured to investigate the recovery effect of stretch motion on the morphological changes of myotube and showed greater value in stretch condition compared (9.87048 ± 0.23759 μm) to the static condition (7.54844 ± 0.19993 μm) (Figure 4a,c). Although there may be various reasons for these results, the reduction of ROS and 8-OHdG is expected to be most closely related to the mitochondrial reduction capacity and NF-Κβ expression. Especially, ROS still remains more than four times the control case but actual DNA damage is lower than control case. It seems stretch encourages enzymes to recover from the DNA damage. The increases in MHC expression and myotube diameter are expected to be associated with PPARγ-related mechanotransduction pathways [38,39].

This study has some limitations worth noting. While we focused on the cyclic stretch to investigate the recovery of damaged muscle cells, several other factors such as cell density, O_2_/CO_2_ concentration, or temperature may be controlled to enhance recovery [40,58]. These variables influence various metabolism including O_2_ consumption of cells, protein synthesis, and pathway expressions, and may be combined with other current works for suggesting innovative therapy of muscle recovery. In addition, the damage confirmation and recovery comparison results only confirm the increase/decrease tendency of DNA damage through 8-OHdG intensity due to the increase/decrease of ROS, but the exact correlation between these parameters has not been elucidated since the relationship between ROS and 8-OHdG is not linear, and the complexity of the biological system is intricately intertwined.

## 5. Conclusions

In conclusion, we have developed a microfluidic system that mimics the strain-damage model and allows in situ generation of damage and recovery of muscle cells to show the constructive effects of cyclic stretch. For the future direction of this research, other meaningful parameters could be investigated to suggest effective treatment to enhance muscle recovery. For example, our platform could be used for in situ application of various treatment types such as cryo-/thermo- therapy, electrotherapy, or hypoxia therapy while monitoring the change in signal of different biomarkers for quantifying the effectiveness of recovery and suggesting innovative treatment strategies for muscle recovery. Furthermore, testing different cyclic stretch ranges and frequencies would reveal the full spectrum of effects that the cyclic stretch has on the recovery of damaged muscle cells for establishing the most effective stretch condition for recovery. Finally, considering that stretch on muscle during rehabilitation shortens the recovery time and increases muscle strength [8,9,10,11], the current work may be further expanded for investigating the optimal degree of stretching during rehabilitation and adding synergistic treatment methods. After accomplishing these further works, we expect that our in situ platform could contribute to developing efficient muscle recovery treatments.

## Supplementary Materials

The supporting materials are available online at http://www.mdpi.com/2072-666X/9/12/671/s1.

## References

[1] Tero A.H., Järvinen M.J., Kalimo H. (2013). Regeneration of injured skeletal muscle after the injury. Muscles Ligaments Tendons J..

[2] Ekstrand J., Hagglund M., Walden M. (2011). Epidemiology of muscle injuries in professional football (soccer). Am. J. Sports Med..

[3] Huard J., Li Y., Fu F.H. (2002). Muscle injuries and repair: Current trends in research. J. Bone Joint Surg. Am. Vol..

[4] Grounds M.D., Yablonka-Reuveni Z., Partridge T. (1993). Molecular and cell biology of skeletal muscle regeneration. Molecular and Cell Biology of Muscular Dystrophy.

[5] Aarimaa V., Rantanen J., Best T., Schultz E., Corr D., Kalimo H. (2004). Mild eccentric stretch injury in skeletal muscle causes transient effects on tensile load and cell proliferation. Scand. J. Med. Sci Sports.

[6] Jarvinen T.A., Jarvinen T.L., Kaariainen M., Kalimo H., Jarvinen M. (2005). Muscle injuries: Biology and treatment. Am. J. Sports Med..

[7] Jankowski R.J., Deasy B.M., Cao B., Gates C., Huard J. (2002). The role of CD34 expression and cellular fusion in the regeneration capacity of myogenic progenitor cells. J. Cell Sci..

[8] Ekstrand J., Askling C., Magnusson H., Mithoefer K. (2013). Return to play after thigh muscle injury in elite football players: Implementation and validation of the Munich muscle injury classification. Br. J. Sports Med..

[9] Järvinen M. (1975). Healing of a crush injury in rat striated muscle: 2. A histological study of the effect of early mobilization and immobilization on the repair processes. Acta Pathol. Microbiol. Scand. Sect. A Pathol..

[10] Valkering K.P., Aufwerber S., Ranuccio F., Lunini E., Edman G., Ackermann P.W. (2017). Functional weight-bearing mobilization after Achilles tendon rupture enhances early healing response: A single-blinded randomized controlled trial. Sports Traumatol..

[11] McCormack R., Bovard J. (2015). Early functional rehabilitation or cast immobilisation for the postoperative management of acute Achilles tendon rupture? A systematic review and meta-analysis of randomised controlled trials. Br. J. Sports Med..

[12] Witard O., Wardle S., Macnaughton L., Hodgson A., Tipton K. (2016). Protein Considerations for Optimising Skeletal Muscle Mass in Healthy Young and Older Adults. Nutrients.

[13] McGlory C., Devries M., Phillips S. (2016). Skeletal muscle and resistance exercise training; the role of protein synthesis in recovery and remodeling. J. Appl. Physiol..

[14] Morelli K.M., Brown L.B., Warren G.L. (2018). Effect of NSAIDs on Recovery from Acute Skeletal Muscle Injury: A Systematic Review and Meta-analysis. Am. J. Sports Med..

[15] Järvinen M., Lehto M., Sorvari T., Mikola A. (1992). Effect of some anti-inflammatory agents on the healing of ruptured muscle: An experimental study in rats. J. Sport Traumatol. Relat. Res..

[16] Rahusen F.T., Weinhold P.S., Almekinders L.C. (2004). Nonsteroidal anti-inflammatory drugs and acetaminophen in the treatment of an acute muscle injury. Am. J. Sports Med..

[17] Beiner J.M., Jokl P., Cholewicki J., Panjabi M.M. (1999). The effect of anabolic steroids and corticosteroids on healing of muscle contusion injury. Am. J. Sports Med..

[18] Carson J.A., Manolagas S.C. (2015). Effects of sex steroids on bones and muscles: Similarities, parallels, and putative interactions in health and disease. Bone.

[19] Levine W.N., Bergfeld J.A., Tessendorf W., Moorman C.T. (2000). Intramuscular corticosteroid injection for hamstring injuries. A 13-year experience in the National Football League. Am. J. Sports Med..

[20] Quattrocelli M., Barefield D.Y., Warner J.L., Vo A.H., Hadhazy M., Earley J.U., Demonbreun A.R., McNally E.M. (2017). Intermittent glucocorticoid steroid dosing enhances muscle repair without eliciting muscle atrophy. J. Clin. Investig..

[21] Menetrey J., Kasemkijwattana C., Day C.S., Bosch P., Vogt M., Fu F.H., Moreland M.S., Huard J. (2000). Growth factors improve muscle healing in vivo. J. Bone Joint Surg. Br. Vol..

[22] Kasemkijwattana C., Menetrey J., Bosch P., Somogyi G., Moreland M.S., Fu F.H., Buranapanitkit B., Watkins S.S., Huard J. (2000). Use of growth factors to improve muscle healing after strain injury. Clin. Orthop. Relat. Res..

[23] Diao Y.-P., Cui F.-K., Yan S., Chen Z.-G., Lian L.-S., Guo L.-L., Li Y.-J. (2016). Nerve Growth Factor Promotes Angiogenesis and Skeletal Muscle Fiber Remodeling in a Murine Model of Hindlimb Ischemia. Chin. Med. J..

[24] Miller K.J., Thaloor D., Matteson S., Pavlath G.K. (2000). Hepatocyte growth factor affects satellite cell activation and differentiation in regenerating skeletal muscle. Am. J. Physiol. Cell. Physiol..

[25] White J., Smythe G. (2016). Growth Factors and Cytokines in Skeletal Muscle Development, Growth, Regeneration and Disease.

[26] Rantanen J., Thorsson O., Wollmer P., Hurme T., Kalimo H. (1999). Effects of therapeutic ultrasound on the regeneration of skeletal myofibers after experimental muscle injury. Am. J. Sports Med..

[27] Markert C.D., Merrick M.A., Kirby T.E., Devor S.T. (2005). Nonthermal ultrasound and exercise in skeletal muscle regeneration. Arch. Phys. Med. Rehabil..

[28] Delgado-Diaz D.C., Gordon B.S., Dompier T., Burgess S., Dumke C., Mazoué C., Caldwell T., Kostek M.C. (2011). Therapeutic Ultrasound Affects IGF-1 Splice Variant Expression in Human Skeletal Muscle. Am. J. Sports Med..

[29] Chongsatientam A., Yimlamai T. (2016). Therapeutic Pulsed Ultrasound Promotes Revascularization and Functional Recovery of Rat Skeletal Muscle after Contusion Injury. Ultrasound Med. Biol..

[30] Koike T., Camargo R.C., Ozaki G., Castoldi R., Seraphim P., Oikawa S., Camargo Filho J.C. (2016). Morphometric and fractal analysis of injured skeletal muscle tissue subjected to a combination of treatments; cryotherapy and therapeutic ultrasound. Int. J. Morphol..

[31] Best T.M., Loitz-Ramage B., Corr D.T., Vanderby R. (1998). Hyperbaric oxygen in the treatment of acute muscle stretch injuries. Results in an animal model. Am. J. Sports Med..

[32] Bennett M., Best T.M., Babul S., Taunton J., Lepawsky M. (2005). Hyperbaric oxygen therapy for delayed onset muscle soreness and closed soft tissue injury. Cochrane Database Syst Rev..

[33] Hyngstrom A.S., Murphy S.A., Nguyen J., Schmit B.D., Negro F., Gutterman D.D., Durand M.J. (2018). Ischemic conditioning increases strength and volitional activation of paretic muscle in chronic stroke: A pilot study. J. Appl. Physiol..

[34] Taylor N.A., Wilkinson J.G. (1986). Exercise-induced skeletal muscle growth. Hypertrophy or hyperplasia?. Sports Med..

[35] Bian W., Bursac N. (2008). Tissue engineering of functional skeletal muscle: Challenges and recent advances. IEEE Eng. Med. Biol. Mag..

[36] Otis J.S., Burkholder T.J., Pavlath G.K. (2005). Stretch-induced myoblast proliferation is dependent on the COX2 pathway. Exp. Cell Res..

[37] Andersen J.I., Juhl M., Nielsen T., Emmersen J., Fink T., Zachar V., Pennisi C.P. (2014). Uniaxial cyclic strain enhances adipose-derived stem cell fusion with skeletal myocytes. Biochem. Biophys. Res. Commun..

[38] Chang Y.J., Chen Y.J., Huang C.W., Fan S.C., Huang B.M., Chang W.T., Tsai Y.S., Su F.C., Wu C.C. (2016). Cyclic Stretch Facilitates Myogenesis in C2C12 Myoblasts and Rescues Thiazolidinedione-Inhibited Myotube Formation. Front. Bioeng. Biotechnol..

[39] Tan J., Kuang W., Jin Z., Jin F., Xu L., Yu Q., Kong L., Zeng G., Yuan X., Duan Y. (2009). Inhibition of NFkappaB by activated c-Jun NH2 terminal kinase 1 acts as a switch for C2C12 cell death under excessive stretch. Apoptosis.

[40] Kim D., Wu X., Young A.T., Haynes C.L. (2014). Microfluidics-based in vivo mimetic systems for the study of cellular biology. Acc. Chem. Res..

[41] Shin Y., Han S., Jeon J.S., Yamamoto K., Zervantonakis I.K., Sudo R., Kamm R.D., Chung S. (2012). Microfluidic assay for simultaneous culture of multiple cell types on surfaces or within hydrogels. Nat. Protoc..

[42] Kim S., Kim W., Lim S., Jeon J. (2017). Vasculature-On-A-Chip for In Vitro Disease Models. Bioengineering.

[43] Bersini S., Jeon J.S., Moretti M., Kamm R.D. (2014). In vitro models of the metastatic cascade: From local invasion to extravasation. Drug Discov. Today.

[44] Madden L., Juhas M., Kraus W.E., Truskey G.A., Bursac N. (2015). Bioengineered human myobundles mimic clinical responses of skeletal muscle to drugs. Elife.

[45] Uzel S.G.M., Platt R.J., Subramanian V., Pearl T.M., Rowlands C.J., Chan V., Boyer L.A., So P.T.C., Kamm R.D. (2016). Microfluidic device for the formation of optically excitable, three-dimensional, compartmentalized motor units. Sci. Adv..

[46] Osaki T., Uzel S.G.M., Kamm R.D. (2018). Microphysiological 3D model of amyotrophic lateral sclerosis (ALS) from human iPS-derived muscle cells and optogenetic motor neurons. Sci. Adv..

[47] Hyun K.U., Sukhee P., Hyunseung B., Mina K., Hyunjun S., Shin J.H. (2018). Promotion of Myogenic Maturation by Timely Application of Electric Field Along the Topographical Alignment. Tissue Eng. Part A.

[48] Agrawal G., Aung A., Varghese S. (2017). Skeletal muscle-on-a-chip: An in vitro model to evaluate tissue formation and injury. Lab Chip.

[49] Huh D., Matthews B.D., Mammoto A., Montoya-Zavala M., Hsin H.Y., Ingber D.E. (2010). Reconstituting Organ-Level Lung Functions on a Chip. Science.

[50] Bächtold P.R. (2009). Stretchable, High–Throughput, Continuously Perfused Cell Culture System.

[51] Lee J.N., Jiang X., Ryan D., Whitesides G.M. (2004). Compatibility of Mammalian Cells on Surfaces of Poly(dimethylsiloxane). Langmuir.

[52] Wan C.R., Chung S., Kamm R.D. (2011). Differentiation of embryonic stem cells into cardiomyocytes in a compliant microfluidic system. Ann. Biomed. Eng..

[53] Apel K., Hirt H. (2004). Reactive oxygen species: Metabolism, oxidative stress, and signal transduction. Annu. Rev. Plant Biol..

[54] Wells L., Edwards K.A., Bernstein S.I. (1996). Myosin heavy chain isoforms regulate muscle function but not myofibril assembly. The EMBO journal.

[55] van der Vliet A., Janssen-Heininger Y.M. (2014). Hydrogen peroxide as a damage signal in tissue injury and inflammation: Murderer, mediator, or messenger?. J. Cell. Biochem..

[56] Kozakowska M., Pietraszek-Gremplewicz K., Jozkowicz A., Dulak J. (2015). The role of oxidative stress in skeletal muscle injury and regeneration: Focus on antioxidant enzymes. J. Muscle Res. Cell Motil..

[57] Brancaccio P., Lippi G., Maffulli N. (2010). Biochemical markers of muscular damage. Clin. Chem. Lab. Med..

[58] Beiner J., Jokl P. (2001). Muscle contusion injuries: Current treatment options. J. Am. Acad. Orthop. Surg..

