# Supplementary material for: Development of Microfluidic Stretch System for Studying Recovery of Damaged Skeletal Muscle Cells
Source: Micromachines (Basel). 2018 Dec 18;9(12):671. doi: 10.3390/mi9120671 (PMC6315523; doi:10.3390/mi9120671)
Supplement: Supplementary file 1 [file micromachines-09-00671-s001.pdf]

Supplementary Materials

Development of Microfluidic Stretch System for Studying Recovery of Damaged Skeletal Muscle Cells

Wanho Kim, Jaesang Kim, Hyung-Soon Park and Jessie S. Jeon

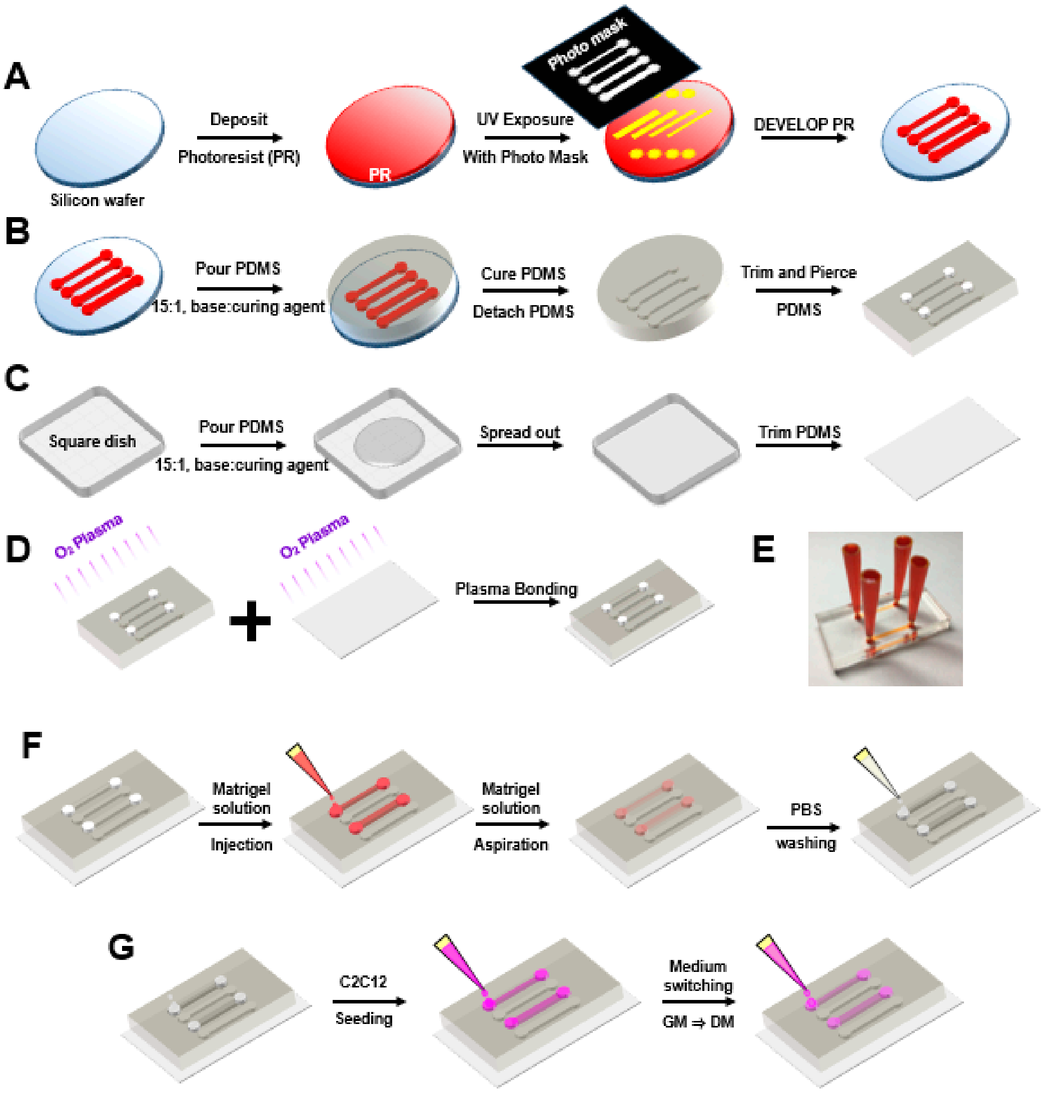

Figure S1. Microfluidic polydimethylsiloxane (PDMS) device manufacturing and cell seeding process. (**a**) Silicon wafer was fabricated using soft lithography method. (**b**) PDMS devices are molded using the silicon wafer. (**c**) PDMS film are made for bonding with PDMS devices to close the channel. (**d**) PDMS device and film were sterilized using an autoclave and later exposed to O_2 plasma for bonding. For strong bonding, the devices are placed in a dry oven (80 °C) for overnight. (**e**) Micropipette tip is recommended to be inserted in the inlet and outlet for reservoir. (**f**) PDMS device bottom surface is coated with Matrigel. (**g**) C2C12 cells are seeded in the PDMS device.

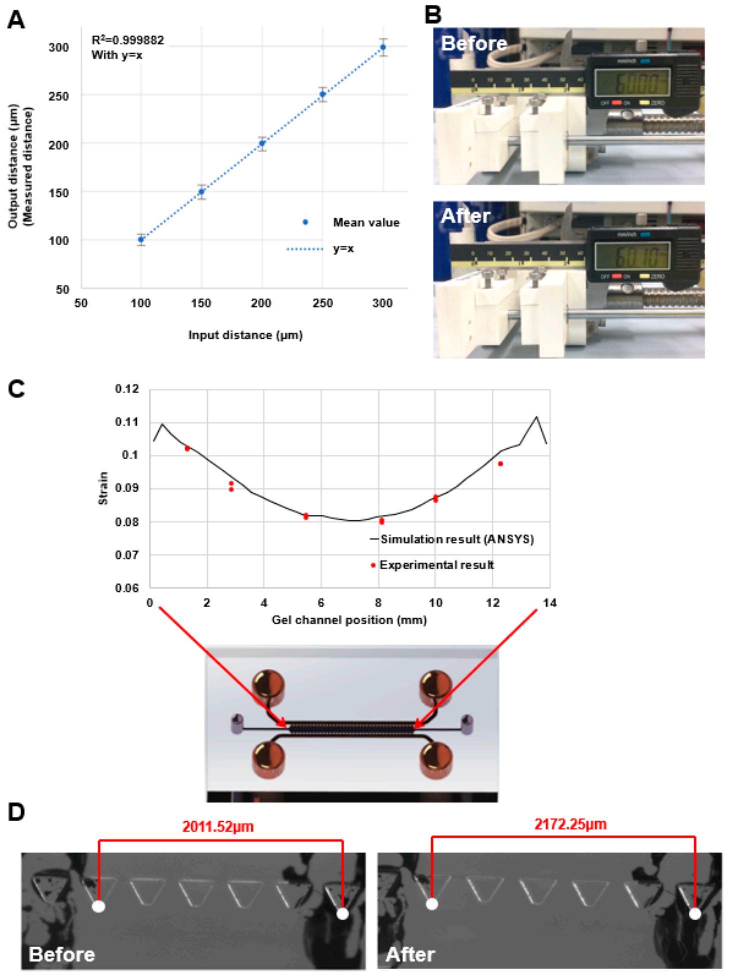

Figure S2. Stretcher accuracy test. (**a**) Control accuracy of stretcher was tested by giving input command of 100, 150, 200, 250, and 300 μm (x-axis) and measuring resultant output distance (y-axis) 32 times. Results show that the stretcher has less than 0.02% positioning error. (**b**) Measurement of output distance is shown for input under 100 μm. (**c**) Simulation and experimental results for strain at different locations of the microfluidic channel are shown when 10% uniaxial stretch is given. (**d**) The distance of channel in microfluidic device is shown for before and after the stretch.

Table S1. Material properties and assumption for ANSYS (ANSYS Inc., Canonsburg, PA, USA) simulation.

Material	Young's Modulus (MPa)	Poisson's Ratio	Assumptions
PDMS [1]	1.26	0.5	Linear, Elastic, Isotropic
PLA [2]	3500	0.36	
Polypropylene (pipette tip) [3]	1300	0.42	

References

1. Shin, H.-J.; Lee, W.-T.; Park, S.-H.; Lee, S.-H.; Park, J.-H.; Yoon, Y.-S.; Shin, J. Development of a tensile cell stimulator to study the effects of uniaxial tensile stress on osteogenic differentiation of bone marrow mesenchymal stem cells. *Trans. Korean Soc. Mech. Eng.* **2009**, *33*, 629–636.
2. Torres, J.; Cotelo, J.; Karl, J.; Gordon, A.P. Mechanical property optimization of FDM PLA in shear with multiple objectives. *Jom* **2015**, *67*, 1183–1193, doi:10.1007/s11837-015-1367-y.
3. Lin, H.; Shi, L.; Wang, D. A rapid and intelligent designing technique for patient-specific and 3D-printed orthopedic cast. *3D Print. Med.* **2015**, *2*, 4.
